# Pregnancy following Radical Resection of Solid Pseudopapillary Tumor of the Pancreas

**DOI:** 10.1155/2014/382535

**Published:** 2014-10-01

**Authors:** James M. O'Brien, Debra Gussman, Ellen Hagopian, Theodore Matulewicz

**Affiliations:** ^1^Department of Obstetrics and Gynecology, Jersey Shore University Medical Center, Neptune, NJ 07753, USA; ^2^Department of General Surgery, Jersey Shore University Medical Center, Neptune, NJ 07753, USA; ^3^Department of Pathology, Jersey Shore University Medical Center, Neptune, NJ 07753, USA

## Abstract

Solid pseudopapillary tumor of the pancreas is a rare tumor seen in predominately young women and carries a low malignant potential. We discuss a patient, who presented to our high risk clinic, with a clinical history of solid pseudopapillary tumor of the pancreas, predating her pregnancy. The patient had undergone previous surgery and imaging which had excluded recurrence of disease; however, increased attention was paid to the patient during her pregnancy secondary to elevated hormonal levels of progesterone, which any residual disease would have a heightened sensitivity to. In cases of pregnant patients with a history of pancreatic tumors, a multidisciplinary approach with maternal fetal medicine, medicine, and general surgery is appropriate and can result in a healthy mother and healthy term infant.

## 1. Introduction

Solid pseudopapillary tumor of the pancreas is considered a rare neoplasm, which predominately affects young African American women [1]. It predominately affects the exocrine function of the pancreas. The origins of the tumor remain unknown, and there are no identifiable risk factors which have been associated with the development of the neoplasm. In addition to its rarity, pseudopapillary tumor of the pancreas is additionally associated with a relatively low malignant potential [2]. Cases of the tumor in pregnancy can be devastating, since the tumor often has receptors which are responsive to progesterone. Even after successful resection of the tumor, attention needs to be paid to patients who subsequently become pregnant, secondary to any residual disease having a heightened sensitivity to increased levels of progesterone.

## 2. Case Report

A 32-year-old G4P3002 female presented for prenatal care. She was treated two years earlier for a solid pseudopapillary tumor of the pancreas. At that time, she presented to the emergency department complaining of lower back pain, early satiety, and nausea associated with meals. CT scan of the abdomen revealed a calcified cystic mass located in the distal portion of the pancreas. Given her symptomatology, the patient underwent an exploratory laparotomy, distal pancreatectomy, and splenectomy.

**Figure 1:**
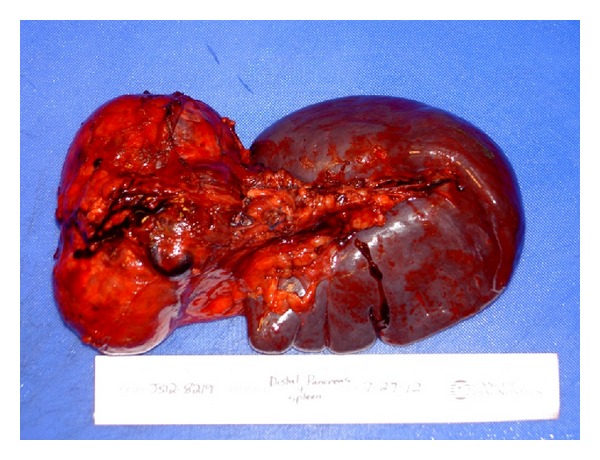
Gross surgical specimen showing the distal pancreas and the spleen.

**Figure 2:**
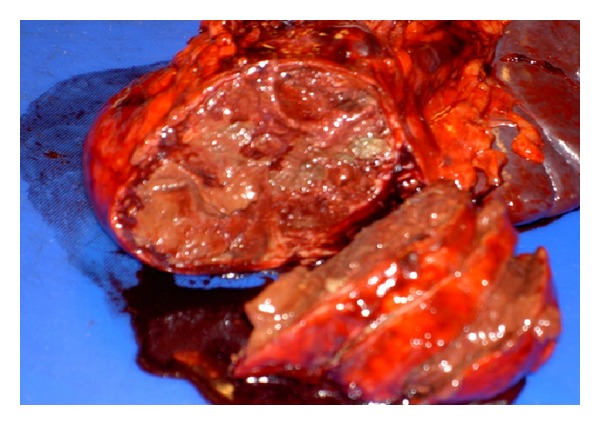
Gross surgical specimen showing a well encapsulated cystic mass located in the distal pancreas.

**Figure 3:**
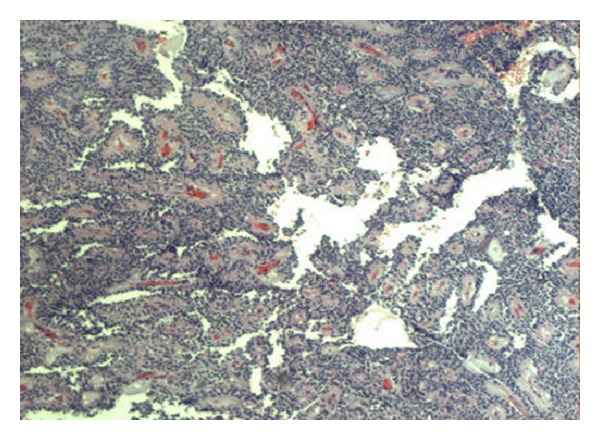
Pathology slide showing pseudopapillae with hyalinized fibrovascular cores.

**Figure 4:**
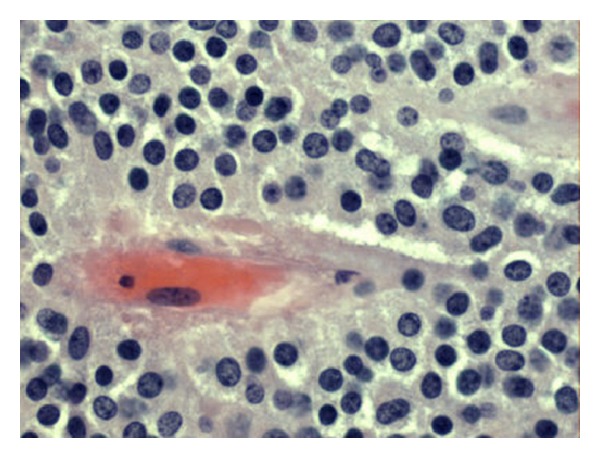
Pathology slide showing hyalinized core, typical of pseudopapillary tumor of the pancreas.

Intraoperative findings showed a large cystic mass of the pancreatic tail, which was adherent to the hilum of the spleen (Figure 1). The patient underwent a distal pancreatectomy and splenectomy. Her postoperative course was uncomplicated. On pathology, a large cystic mass was attached to the slpeen, measuring 10 × 8 × 7 cm (Figure 2). Pathology confirmed the lesion to be solid pseudopapillary neoplasm of the pancreas with clear margins (Figures 3 and 4). She was followed up in surgery clinic and underwent a CT of the abdomen and pelvis one year later which did not demonstrate any evidence of residual or metastatic disease. The patient was deemed as being cured.

From the onset of presenting with her pregnancy, her care was managed with input from maternal fetal medicine, endocrinology, and general surgery. The patient's antepartum care was uncomplicated. She did not report any abdominal pain or diarrhea. She had normal weight gain during her pregnancy and had normal laboratory values, including fasting glucose levels ranging from 72 to 91 mg/dL and a normal 1-hour glucose tolerance test. Liver function tests, amylase, and lipase were additionally normal. The patient underwent a repeat low transverse cesarean section and bilateral tubal ligation at 39 weeks of gestation. Her surgery was uncomplicated and yielded a live born female infant, weighing 3170 grams, with APGARs of 9 and 9 at 1 and 5 minutes, respectively. There was no evidence of residual disease at the time of surgery.

## 3. Discussion

Solid pseudopapillary tumor of the pancreas is a rare neoplasm of the pancreas, predominately affecting the exocrine function of the pancreas. The tumor is most commonly seen in young African American women and has a low malignant potential [1, 2]. It constitutes approximately 1% of all pancreatic tumors and 3% of cystic neoplasms of the pancreas. The rarity of this tumor makes it difficult to differentiate tumors that have a malignant potential from benign before surgical intervention [3]. There is a male to female ratio of 1 : 9.5, with a documented age range of eight to sixty-five years of age [2]. The etiology of the tumor continues to remain a mystery without any identifiable risk factors. Recent reports have described mutations in the beta-catenin oncogene in nearly all of the solid pseudopapillary tumors studied, while Cyclin D1, a downstream transcriptional target of beta-catenin, has been found to be overexpressed in most cases [2].

The presenting signs and symptoms of solid pseudopapillary tumor are often vague. Common complaints are upper abdominal pain, which often radiates to the back [3]. Symptoms of a pancreatic mass, including abdominal discomfort and nausea, may be interpreted as normal symptoms of pregnancy, thus leading to a delay in diagnosis in those presenting during pregnancy [4]. Our patient presented prior to her pregnancy, with typical symptoms including back pain and early satiety associated with nausea. Her tumor was discovered incidentally on a CT scan performed in the emergency department.

Pregnancy results in profound changes in maternal metabolism and insulin secretion, most notably during the last trimester of pregnancy [5]. Pregnancy results in higher *β* cell sensitivity to glucose, increased insulin biosynthesis, and important modifications of the architecture of the islets with *β* cell hyperplasia and hypertrophy [5]. Human placental lactogen hormones trigger the endocrine changes which occur in the pancreas during pregnancy. In terms of exocrine function, the basal secretion of bicarbonate is increased during pregnancy. Furthermore, resting enzyme output is increased during pregnancy. In particular, the response to cholecystokinin during pregnancy is profoundly increased [6].

Pancreatic neoplasms are uncommon during pregnancy. There have been only eight reported cases of pancreatic adenocarcinoma, thirteen cases of cystic pancreatic lesions, and three cases of neuroendocrine tumors [3]. While there are reported cases in the literature addressing cases of solid pseudopapillary tumor of the pancreas during the antepartum and postpartum period, there is a lack of literature regarding patients who have previously been diagnosed and undergone surgical intervention (Table 1). Imaging via ultrasound, CT scan, or MRI remains the mainstay of diagnosis since serum markers such as CA 19-9, CA-50, alpha fetal protein, CEA, CA 125, CA 72-4, and CA 242 are notoriously within normal limits [4]. Cases discovered during the antepartum period during the second trimester benefit from surgical resection with clear margins, which is considered to be curative.

Subsequent pregnancies after diagnosis and resection carry concerns regarding appropriate weight gain during the pregnancy secondary to the tumor predominately affecting the exocrine function of the pancreas. There is the possibility of recurrence during pregnancy secondary to the profound stimulus that progesterone could serve on any residual cancerous tissue. However, this concern should be minimalized if there is pathology confirming clear borders from the time of surgery.

In conclusion, these patients require the attention of a multidisciplinary team of specialists in order to ensure successful pregnancy outcomes. Nutritional consultation can aid in appropriate dietary supplementation for patients who may have compromised exocrine function. Endocrinology was involved in patient care, especially in situations where the endocrine function had been compromised, in order to guarantee appropriate glycemic control. Furthermore, general surgery, preferably the surgeon responsible for the patient's pancreatic resection, should be involved in patient care. This proved particularly valuable in our patient who underwent a repeat cesarean section as her delivery mode, which granted the opportunity to confirm no visible recurrence of disease.

## Figures and Tables

**Table 1:** Cases of pseudopapillary tumor of the pancreas complicating pregnancy.

Time of diagnosis with regard to pregnancy	Maternal outcome	Fetal outcome
Postpartum	Surgery after vaginal delivery	Live born term infant
Antepartum	Surgery at 16 weeks	Live born term infant via vaginal delivery
Antepartum	Surgery at 14 weeks	Live born preterm infant via vaginal delivery
Antepartum	Surgery at 6 weeks	Live born term infant via vaginal delivery
Antepartum	Surgery at 19 weeks	Live born preterm infant via vaginal delivery
Antepartum	Surgery at 23 weeks	Live born term infant via vaginal delivery
Antepartum	Surgery at 13 weeks	Live born term infant via cesarean section

## References

[1] Levy C, Pereira L, Dardarian T, Cardonick E (2004). Solid pseudopapillary pancreatic tumor in pregnancy: a case report. *Journal of Reproductive Medicine for the Obstetrician and Gynecologist*.

[2] Breizat AH, Al-Tahieneh A (2010). Solid pseudopapillary tumor of the pancreas in a pregnant female: A case report. *Journal of Royal Medical Services*.

[3] Boyd CA, Benarroch-Gampel J, Kilic G, Kruse EJ, Weber SM, Riall TS (2012). Pancreatic neoplasms in pregnancy: diagnosis, complications, and management. *Journal of Gastrointestinal Surgery*.

[4] Huang S-C, Wu T-H, Chen C-C, Chen T-C (2013). Spontaneous rupture of solid pseudopapillary neoplasm of the pancreas during pregnancy. *Obstetrics and Gynecology*.

[5] Bernard-Kargar C, Ktorza A (2001). Endocrine pancreas plasticity under physiological and pathological conditions. *Diabetes*.

[6] Rosenberg V, Rudick J, Robbiou M, Dreiling DA (1975). Pancreatic exocrine secretion during and after pregnancy. *Annals of Surgery*.

